# Astragaloside IV inhibits astrocyte senescence: implication in Parkinson’s disease

**DOI:** 10.1186/s12974-020-01791-8

**Published:** 2020-04-06

**Authors:** Mei-Ling Xia, Xia-Hong Xie, Jian-Hua Ding, Ren-Hong Du, Gang Hu

**Affiliations:** 1 Department of Pharmacology, School of Medicine and Life Sciences, Nanjing University of Chinese Medicine, 138 Xianlin Avenue, Nanjing, Jiangsu 210023 People’s Republic of China; 2 Jiangsu Key Laboratory of Neurodegeneration, Department of Pharmacology, Nanjing Medical University, 101 Longmian Avenue, Nanjing, Jiangsu 211166 People’s Republic of China

**Keywords:** Astragaloside IV, Astrocyte, Cell senescence, Mitophagy, Parkinson’s disease

## Abstract

**Background:**

Senescent astrocytes have been implicated in the aging brain and neurodegenerative disorders, including Parkinson’s disease (PD). Astragaloside IV (AS-IV) is an antioxidant derivative from a traditional Chinese herbal medicine Astragalus membraneaceus Bunge and exerts anti-inflammatory and longevity effects and neuroprotective activities. However, its effect on astrocyte senescence in PD remains to be defined.

**Methods:**

Long culture-induced replicative senescence model and lipopolysaccharide/1-methyl-4-phenylpyridinium (LPS/MPP^+^)-induced premature senescence model and a mouse model of PD were used to investigate the effect of AS-IV on astrocyte senescence in vivo and in vitro. Immunocytochemistry, qPCR, subcellular fractionation, flow cytometric analyses, and immunohistochemistry were subsequently conducted to determine the effects of AS-IV on senescence markers.

**Results:**

We found that AS-IV inhibited the astrocyte replicative senescence and LPS/MPP^+^-induced premature senescence, evidenced by decreased senescence-associated β-galactosidase activity and expression of senescence marker p16, and increased nuclear level of lamin B1, and reduced pro-inflammatory senescence-associated secretory phenotype. More importantly, we showed that AS-IV protected against the loss of dopamine neurons and behavioral deficits in the mouse model of PD, which companied by reduced accumulation of senescent astrocytes in substantia nigra compacta. Mechanistically, AS-IV promoted mitophagy, which reduced damaged mitochondria accumulation and mitochondrial reactive oxygen species generation and then contributed to the suppression of astrocyte senescence. The inhibition of autophagy abolished the suppressive effects of AS-IV on astrocyte senescence.

**Conclusions:**

Our findings reveal that AS-IV prevents dopaminergic neurodegeneration in PD via inhibition of astrocyte senescence through promoting mitophagy and suggest that AS-IV is a promising therapeutic strategy for the treatment of age-associated neurodegenerative diseases such as PD.

## Background

Parkinson’s disease (PD), a common neurodegenerative disease, is characterized by the dopaminergic (DA) neuron death in the substantia nigra compacta (SNc) [1]. Although the mechanisms of PD have not still been clearly clarified, emerging evidence suggests that astrocytes have been involved in the pathogenesis of PD [2]. Astrocytes are the most populous glial subtype and are critical for brain function. They regulate ion balance and provide metabolic and neurotrophic support in the central nervous system (CNS). Therefore, the disruption of astrocyte biology may contribute to CNS dysfunction and pathology [3].

Aging is the most crucial risk factor for the development of PD [4]. Studies have shown that the annual incidence of PD increases exponentially with age. It is, therefore, possible that cell senescence contributes to its pathophysiology [5]. Cellular senescence is one of the essential mechanisms of aging and has been recognized as a central component of age-associated neurodegenerative disorders [6]. Senescent cells increase the secretion of proteases, pro-inflammatory cytokines, and chemokines, known as senescence-associated secretory phenotype (SASP), which strengthens cell senescence and spreads this phenotype to surrounding cells in a paracrine and autocrine way [7, 8]. Increasing evidence indicates the cellular senescence that plays a profound role in the initiation and progression of neurodegenerative diseases such as PD and cognitive decline with aging [4] [9–11]. Therefore, targeting senescent astrocytes could represent a novel approach toward therapies for age-associated neurodegenerative diseases [12].

Astragaloside IV (AS-IV) is purified from the Chinese medicinal herb, Astragalus membranaceus. AS-IV has been reported to exert anti-aging and immunomodulatory effects, and anti-inflammatory and anti-oxidative neuroprotective activities [13–16]. Previously, AS-IV was reported to reduce apoptosis and increase the resistance of dopamine neurons to neurotoxins in vitro [17]. Nevertheless, the effects of AS-IV on astrocyte function in PD have not yet been investigated. Given the importance of astrocyte senescence in PD, we hypothesize that AS-IV may inhibit astrocyte senescence from preventing DA neurodegeneration in the pathogenesis of PD. In the present study, we demonstrated that AS-IV significantly protected mice against neurodegeneration in the murine PD model through inhibiting senescent astrocytes. Further study revealed that AS-IV triggered mitophagy and then reduced the accumulation of dysfunctional mitochondria and mitochondrial reactive oxygen species (ROS) production, thereby inhibiting the astrocyte senescence. The findings obtained in this study may help direct clinical decisions regarding the use of AS-IV in age-associated neurodegenerative diseases such as PD.

## Methods

### Experimental animals

Mice were maintained and bred in the Animal Resource Centre of the Faculty of Medicine, Nanjing Medical University. Mice are free to access to food and water in a room with an ambient temperature of 22 °C ± 2 °C and a light/dark cycle of 12:12 h. All animal procedures were carried out in strict accordance with the guideline of the Institutional Animal Care and Use Committee of Nanjing Medical University.

### 1-Methyl-4-phenyl-1,2,3,6-tetrahydropyridine (MPTP)-induced PD mouse model

MPTP/ probenecid mouse model is used widely for preclinical in vivo assessment of anti-parkinsonian effects, due to its high reproducibility and disease-related pathological features. MPTP is first taken up by astrocytes and converted there to the toxic metabolite 1-methyl-4-phenylpyridinium (MPP^+^) by the MAO enzyme. MPP^+^ is then released and selectively taken up to the dopaminergic nerve terminals by the dopamine transporters and causes the oxidative damage of dopaminergic neurons. Probenecid serves to elevate concentrations of MPTP in the brain by reducing renal elimination of the MPTP. Mice (male, aged 4–5 months old) were randomly divided into saline-treated group, MPTP-treated group, MPTP + AS-IV-treated group, and AS-IV alone treated group. In the MPTP-treated group, mice received subacute MPTP administration. MPTP (Sigma, St. Louis, MO, USA) was injected subcutaneously at 30 mg kg^−1^, followed by 250 mg/kg probenecid (Sigma, St. Louis, MO, USA) at intervals of 1 h for 5 consecutive days and left for 3 days. The mice received chronic MPTP administration as follows: mice were subcutaneously injected with 20 mg/kg MPTP and then intraperitoneally injected with 250 mg/kg probenecid at intervals of 1 h every 3.5 days for 5 weeks. Control mice were treated with saline and probenecid. In the MPTP + AS-IV-treated group, mice were administered with an intraperitoneal injection of 100 mg kg^−1^ AS-IV (Jingzhu Biotechnology, Nanjing, China) at 12-h intervals before MPTP injection. One week after the last injection, mice were anesthetized with 40 mg kg^−1^ sodium pentobarbital injection (Sigma, St. Louis, MO, USA). Brain tissues were homogenized for qPCR analysis and western blotting or were sectioned for immunohistochemistry.

### Behavioral analysis

One week after the final injection of MPTP, the pole test, traction test, and rotarod test were performed. For the pole test, place the mouse head up on the top of a vertical wooden rough-surfaced pole (height 50 cm, diameter 1 cm). Each mouse was accustomed to the apparatus 1 day before testing. Record the total time (T-total, until the mouse reached the floor with its four paws) and the turn time (T-turn, for the mouse to turn completely head downward). The mouse was allowed to descend three times, and the best performance for the T-turn and T-total was obtained. If the mouse did not turn entirely downwards, fell, or slipped down, the default value of 120 s was considered. For the traction test, place the mouse on a rope (diameter 5 mm), and the scores range from 0 to 4, based on its limb placements. The score was evaluated according to the following criteria: if four limbs seized the rope and moved to the side, the score was 4, and if four limbs seized the rope but could not move to the side, the score was 3. If the two or one front limbs seized the rope, the score was 2 or 1, respectively. If the mouse slipped down, the score was 0. In addition, the mice were allowed to hang upside down. For the rotarod test, mice were accustomed to the rotarod (5 min with a fixed speed of 12 rpm) for 3 days before the start of the experiment and then placed the mice on the rod and tested at 20 rpm for 5 min and recorded the latency time which each animal could stay on the rod at test speed. The tester was blinded to all genotypes and treatment groups for each behavioral testing.

### Immunohistochemical analysis and immunofluorescence

The immunostaining method was described in the previous publication [1]. To detect tyrosine hydroxylase (TH), the sections were incubated with mouse monoclonal antibody against TH (T1299, 1:1000, Sigma, St Louis, MO, USA) and then for 1 h with anti-mouse IgG, HRP-linked secondary antibodies (7076, 1:5000, Cell Signaling Technology, USA). Immunoreactivity was visualized by incubation in the substrate-chromogen solution (DAB). Control staining was performed without primary antibodies. The total number of TH-positive neurons in the SNc was recorded stereologically using the optical fractionator method with the MicroBrightField Stereo-Investigator software (MicroBrightField, Williston, VT, USA).

For immunofluorescence staining, the sections were blocked with 10% bovine serum albumin (BSA) in Tris-buffered saline (TBS) + 0.05% Triton X-100 for 1 h at 20 °C. Next, the sections were incubated with the following primary antibodies at 4 °C overnight: anti-glial fibrillary acidic protein (GFAP, Millipore, MAB360; 1:800 dilution), anti-lamin B1 (Abcam, ab16048, 1:200 dilution), and anti-p16 (Santa Cruz Biotechnology, sc-56330, 1:200 dilution). After that, the sections were incubated with Alexa Fluor 488-conjugated or Alexa Fluor 555-conjugated secondary antibody for 1 h at 20 °C. Then, the sections were washed and mounted onto glass slides. DAPI (Life Technologies, P36931) was used to visualize nuclei. Images were taken under a confocal microscope (Axiovert LSM510, Carl Zeiss Co., Germany).

### Primary astrocyte culture and treatment

Astrocyte culture was described in the previous publication [18]. Briefly, the neonatal midbrain is trypsinized with 0.25% trypase (Amresco, Solon, OH) at 37 °C and dissociated, and the cells were plated on a poly-lysine-treated culture plates in Dulbecco’s modified Eagle’s medium (DMEM)/Ham’s F12 medium containing 10% fetal bovine serum (FBS, GIBCO, Gaithersburg, MD, USA). After 24 h, the culture media were changed to complete medium. The purity of astrocytes was > 95%, as determined with GFAP immunocytochemistry. Astrocytes were pretreated with AS-IV for 1 h before MPP^+^ (200 μM, Sigma, St. Louis, MO, USA) or lipopolysaccharide (LPS, 500 ng/ml, Sigma, St. Louis, MO, USA) stimulation for 24 h.

To induce replicative cellular senescence, astrocytes were cultured for 40 days in vitro (DIV) and passaged every 4–5 days (passages 8–10) until their growth slowed to a point at which the cell number in the culture remained stationary (Fig. s1), and then treated with AS-IV at the indicated concentration for 10 days. Control group astrocytes were cultured for 10 days in vitro and then treated with AS-IV at the indicated concentration for 10 days. For pharmacological measurement, autophagy inhibitor 3-MA (3 mM, Tocris, UK) was added to the cell culture medium 1 h before AS-IV treatment. The cell extracts were analyzed by qPCR, flow cytometry, and immunoblotting.

### β-Galactosidase staining

Astrocytes seeded on chamber slides were washed with PBS and fixed in 4% paraformaldehyde in PBS for 30 min at 20 °C. Astrocyte senescence was evaluated using β-galactosidase-based Senescence Cells Staining Kit (CS0030-1KT, Sigma-Aldrich, USA) according to the manufacturer’s instructions. Astrocytes stained blue at pH 6 were observed by a light microscope. The astrocyte nuclei were stained with DAPI. The number of positively stained cells and the number of total cells number were counted. Positive cells are expressed as a percentage of total cell number. Data were obtained from three different cultures.

### Cell viability assay

According to the manufacturer’s instructions, Cell viability was measured by Cell Counting Kit-8 (CCK-8, Biotool, Houston, TX). Briefly, 4 × 10^4^ cells per well were seeded in a 96-well plate and then were treated with AS-IV (0-200 μM) for 10 days. After that, 10 μl of CCK-8 reagent was added to each well for 4 h. Finally, the absorbance was measured at 450 nm using a multi-well spectrophotometer (Bio-Rad). The cell viability of AS-IV-treated astrocytes was described as a percentage compared with control cells, and the cell viability of control cells was considered to be 100%.

### Reverse transcription and quantitative real-time PCR

Trizol reagent (Invitrogen, USA) was used to extract total RNA from SNc tissues and cultured astrocytes. Reverse transcription PCR was performed using a TAKARA PrimeScript RT reagent kit. Then, real-time PCR was measured using a QuantiTect SYBR Green PCR kit (Qiagen, Germany) with an ABI 7300 Fast Real-Time PCR System (Applied Biosystems, Foster City, CA, USA). GAPDH was used as an internal control for the real-time PCR amplification. The sequences of primers for real-time PCR analysis are as follows: GAPDH forward: CAAAAGGGTCATCTCC, reverse: CCCCAGCATCAAAGGTG. p16^Ink4a^ forward: CGCTTCTCACCTCGCTTGT, reverse: TGACCAAGAACCTGCGACC. IL-1β forward: TCATTGTGGCTGTGGAGAAG, reverse: AGGCCACAGGTATTTTGTCG. IL-1α forward: AGTCAACTCATTGGCGCTTG, reverse: GAGAGAGATGGTCAATGGCAGA. IL-6 forward: TCCTTCCTACCCCAATTTCCA, reverse: GTCTTGGTCCTTAGCCACTCC. Cxcl-1 forward: TGCACCCAAACCGAAGTCAT, reverse: CTCCGTTACTTGGGGACACC. MMP-3 forward: GTTCTGGGCTATACGAGGGC, reverse: TTCTTCACGGTTGCAGGGAG. MMP-9 forward: CGACTTTTGTGGTCTTCCCC, reverse: AGCGGTACAAGTATGCCTCTG.

### Measurement of mitochondrial ROS and mitochondrial membrane potential

Mitochondrial ROS and mitochondrial membrane potential methods were described in the previous publication [1]. Astrocytes were stained with MitoSOX (2.5 μM, Invitrogen, USA) or with JC1 (10 μg/ml, T-3168, Invitrogen, USA) at 37 °C for 30 min. After washing cells with PBS twice, the cells were then resuspended in cold PBS containing 1% FBS for flow cytometric analyses. The data were analyzed with the FCS Express software (Guava Easy Cyte™8, Millipore, USA).

### Mitochondrial isolation

Mitochondrial extraction kit (Solarbio, China) was used to isolate astrocyte mitochondria. Astrocytes were homogenized in ice-cold lysis buffer with a Dounce-type glass homogenizer and centrifuged at 600*g* for 10 min. The crude supernatant was then centrifuged for 10 min at 11,000*g* to pellet the intact mitochondria. After that, the pellet was homogenized in 50 μl lysis buffer with 1 μl cocktail (Roche). Then, the homogenate was spun (1000×*g* for 10 min at 4 °C) for three times to precipitate nuclei and tissue debris, yielding a supernatant enriched in mitochondria. Mitochondria from the supernatant were finally precipitated by centrifugation at 16,000×*g* at 4 °C for 15 min.

### Western blotting analysis

Cell lysates and SNc tissues were homogenized in RIPA lysis buffer, and protein concentration was determined by the Bradford assay (Bio-Rad, Hercules, CA, USA). A 30-μg protein aliquot of each sample was separated using standard SDS-PAGE and transferred onto a PVDF membrane (Millipore, Bedford, MA). Immunoreactive bands were detected by enhanced chemiluminescence plus detection reagent (Pierce, Rockford, IL) and analyzed using the ImageQuant™ LAS 4000 imaging system (GE Healthcare, Pittsburgh, PA, USA). The following primary antibodies were used: anti-TH (T1299, Sigma, St Louis, MO, USA), anti-p16 (sc-56330, Santa Cruz Biotechnology, USA), anti-β-actin (BM0627, Boster, Pleasanton, CA, USA), anti-LC3 (3868, Cell Signaling Technology, USA), anti-p62 (23214, Cell Signaling Technology, USA), anti-COX IV (4844, Cell Signaling Technology, USA), anti-TOM20 (42406, Cell Signaling Technology, USA), anti-Parkin (ab77924, Abcam, USA), and anti-PINK1 (ab23707, Abcam, USA).

### Statistical analysis

All data are expressed as means ± SEM. The differences with different treatments were determined by one-way or two-way ANOVA, followed by the Tukey’s post hoc test, and were considered as statistically significant at *p* < 0.05.

## Results

### AS-IV rescues MPTP-induced the loss of DA neurons and behavioral deficits in mice

**Fig. 1:**
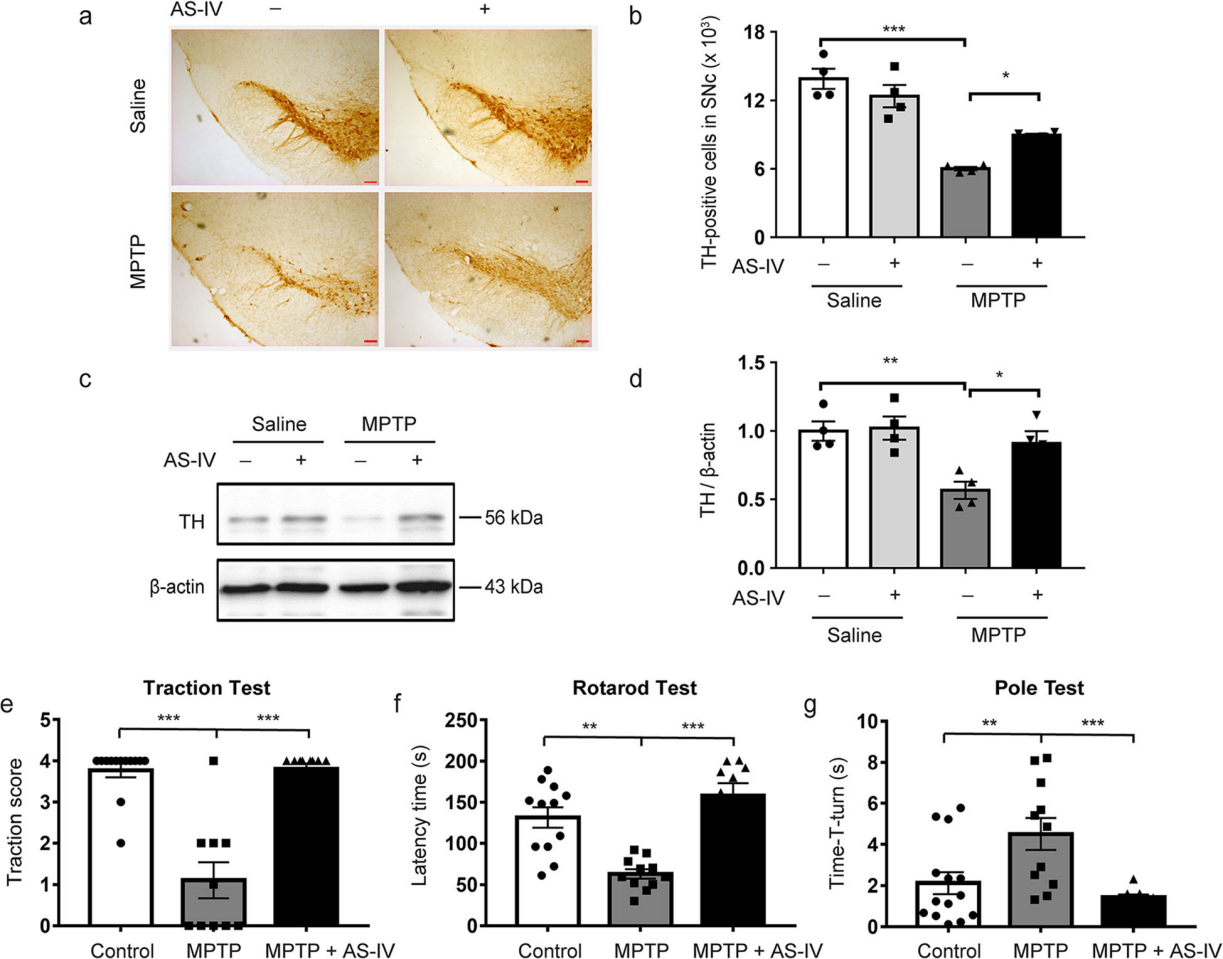
AS-IV rescues MPTP-induced the loss of DA neurons and behavioral deficits in mice. **a** Microphotographs of tyrosine hydroxylase (TH)-positive neurons in the substantia nigra compacta (SNc). **b** Stereological counts of TH-positive neurons in the SNc. **c** Western blotting analysis of TH in the SNc. **d** Quantitative analysis of TH in the SNc. Quantified data are normalized to the control group (the control group value is equal to 1). The data shown are the mean ± SEM. ^*^p < 0.05, ^**^p < 0.01, ^***^p < 0.001. *n* = 4 for each group. Scale bar 100 μm. **e** Traction score in the traction test. **f** Time on the rod in the rotarod test. **g** T-turn time in the pole test. The data shown are the mean ± SEM. ^**^p < 0.01, ^***^p < 0.001. *n* = 8–14. AS-IV, Astragaloside IV; MPTP, 1-methyl-4-phenyl-1, 2, 3, 6-tetrahydropyridine

The neuroprotective of AS-IV was evaluated in the MPTP model of PD. MPTP injection induces a significant loss of TH in the SNc, which is prevented by AS-IV (Fig. 1a, b). Western blot analysis reveals that MPTP-mediated reduction in TH protein expression is restored by AS-IV in the SNc (Fig. 1c, d). The traction test is utilized to measure muscle strength and equilibrium. There is a marked lower traction score in the MPTP-treated mice, which is prevented by AS-IV (Fig. 1e). AS-IV also significantly reduces the behavioral deficits elicited by MPTP injected as measured by the accelerating rotarod test and the pole test (Fig. 1f, g) without affecting MPTP metabolism (Fig. s2). These data indicate that AS-IV rescues MPTP-induced PD like pathology in mice.

### AS-IV reduces the accumulation of senescent astrocytes in SNc of MPTP PD mice

**Fig. 2:**
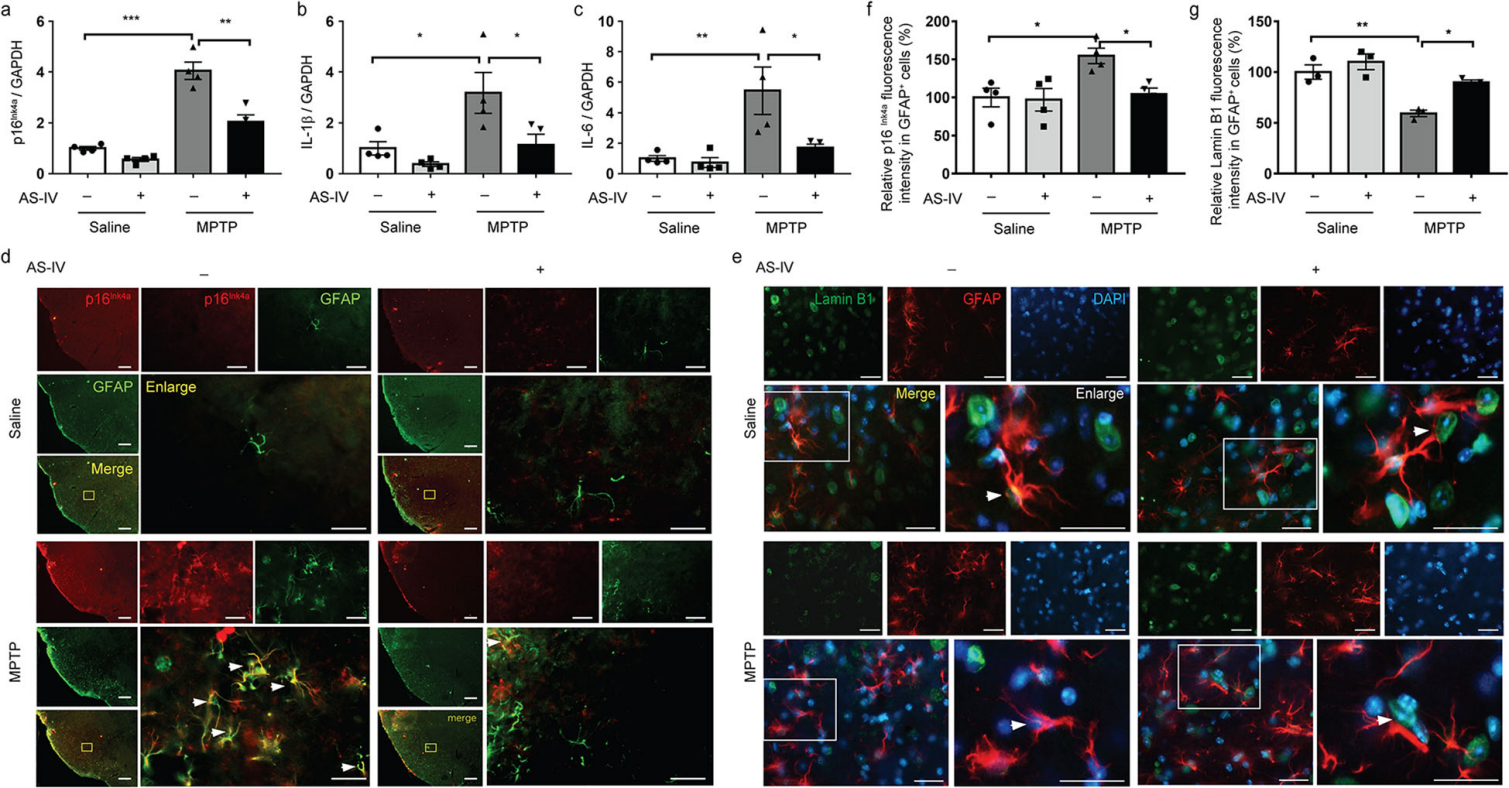
AS-IV reduces senescence markers in astrocytes of the MPTP PD model. **a**–**c** RNA isolated from the substantia nigra compacta (SNc) tissues were analyzed for p16^Ink4a^ (**a**) and SASP factor IL-1β (**b**) and IL-6 (**c**) mRNA levels by qPCR. Quantified data are normalized to the control group (the control group value is equal to 1). The data shown are the mean ± SEM. ^*^p < 0.05, ^**^p < 0.01, ^***^p < 0.001. *n* = 5 for each group. **d** Representative immunofluorescence images showing p16^Ink4a^ protein levels (red) in GFAP^+^ astrocytes (green) in the SNc. Scale bar 200/25 μm. **e** Representative immunofluorescence images showing lamin B1 protein levels (green) in GFAP^+^ astrocytes (red) in the SNc. Scale bar 25 μm. **f** Quantification of relative p16^Ink4a^ fluorescence intensity in GFAP^+^ astrocytes (*n* = 4 for each group). **g** Quantification of relative lamin B1 fluorescence intensity in GFAP^+^ astrocytes (*n* = 3 for each group). Quantified data are normalized to the control group (the control group value is equal to 100%). The data shown are the mean ± SEM. ^*^p < 0.05, ^**^p < 0.01. AS-IV, Astragaloside IV; MPTP, 1-methyl-4-phenyl-1, 2, 3, 6-tetrahydropyridine

Increasing evidence indicates that senescent astrocytes play important roles in PD [11], prompting us to ask whether AS-IV could attenuate senescent astrocytes in SNc of MPTP model mice. Compared to control mice, the MPTP-injected mice displayed elevated expression of the senescence marker p16^Ink4a^, and several SASP factors included the pro-inflammatory cytokines interleukin-6 (IL-6), and IL-1β, which greatly decreased by the AS-IV treatment (Fig. 2a–c). We next determined whether AS-IV inhibited astrocyte senescence in PD. Remarkably, a significant population of senescent astrocytes overexpressing p16 is present in SNc of MPTP model mice, and the AS-IV treatment inhibited this MPTP-induced astrocyte senescence (Fig. 2d, f). In addition, a reduced nuclear level of lamin B1, an established senescence-associated marker, was found in astrocytes in SNc of MPTP model mice, and the AS-IV significantly suppressed the downregulation of nuclear lamin B1 (Fig. 2e, g). These results demonstrate that AS-IV decreases the accumulation of senescent astrocytes in SNc of MPTP PD mice.

### AS-IV inhibits long-term culture-induced replicative senescence in astrocytes

**Fig. 3:**
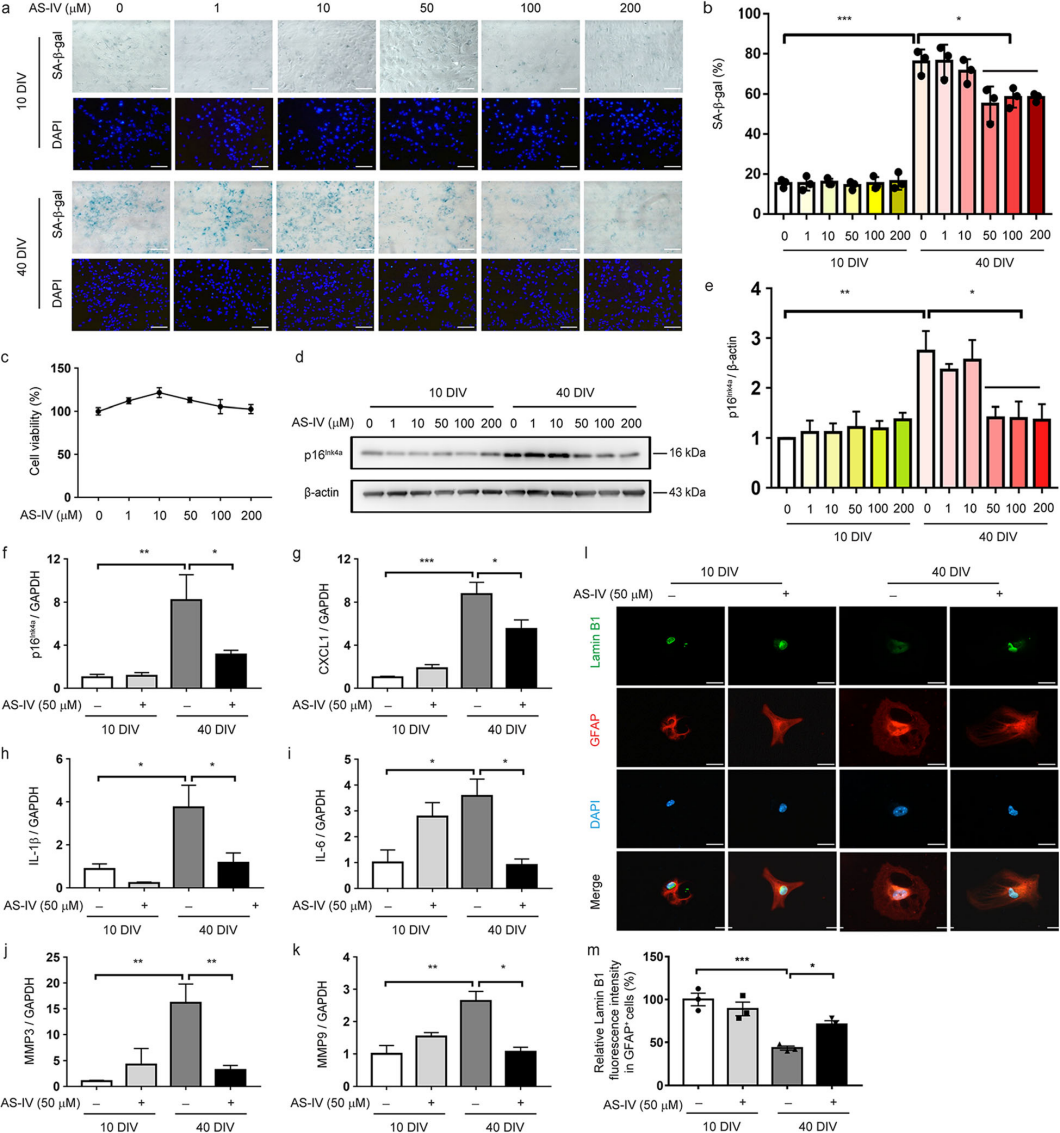
AS-IV inhibits senescent phenotypes in cultured astrocytes. Astrocytes were cultured for 10 days or 40 days in vitro (DIV) and then treated with AS-IV (1, 10, 50, 100, 200 μM) for 10 days. **a**, **b** Representative images of SA-β-gal activity (**a**) and percentage of SA-β-gal^+^ cells (**b**) (from three independent experiments). DAPI staining nucleus (blue). Scale bar 100 μm. **c** Cell viability was measured by CCK-8 assay (from five independent experiments). **d**, **e** Representative immunoblots (**d**) and quantitative analysis of p16^Ink4a^ (**e**) in astrocytes (from four independent experiments). **f**–**k** qPCR of p16^INK4a^ (**f**), CXCL1 (**g**), IL-1β (**h**), IL-6 (**i**), MMP3 (**j**), and MMP9 **(k**) mRNA levels (from four to six independent experiments). Quantified data are normalized to the control group (the control group value is equal to 1). The data shown are the mean ± SEM. ^*^p < 0.05, ^**^p < 0.01, ^***^p < 0.001. **l** Representative images of lamin B1 immunofluorescence (green) in GFAP^+^ astrocytes (red). **m** Quantification of relative lamin B1 fluorescence intensity in GFAP^+^ astrocytes. Quantified data are normalized to the control group (the control group value is equal to 100%). The data shown are the mean ± SEM from three independent experiments. ^*^p < 0.05, ^***^p < 0.001. DAPI staining nucleus (blue). Scale bar 25 μm. AS-IV, Astragaloside IV

Next, we examined the direct effect of AS-IV on astrocyte senescence in vitro. To evaluate the role of AS-IV on replicative senescence, mouse astrocytes made senescent by in vitro culturing for 40 days, showed signs of a senescence phenotype, including elevated senescence-associated β-galactosidase, and increased expression of senescence marker p16, which were indeed inhibited by AS-IV in a concentration-dependent manner without affecting cell survival (Fig. 3a–e). Since AS-IV induced a substantial suppressive effect at 50 μM concentration, we used this concentration in the subsequent studies. Also, compared to nonsenescent astrocytes, senescent astrocytes displayed elevated expression of the several SASP factors and reduced the nuclear level of lamin B1. AS-IV treatment inhibited the increase in SASP factors and a decrease in lamin B1 in senescent astrocytes (Fig. 3f–m). These findings indicate that AS-IV inhibits the replicative senescence of astrocytes.

### AS-IV suppresses MPP^+^/LPS-induced premature senescence of astrocytes

**Fig. 4:**
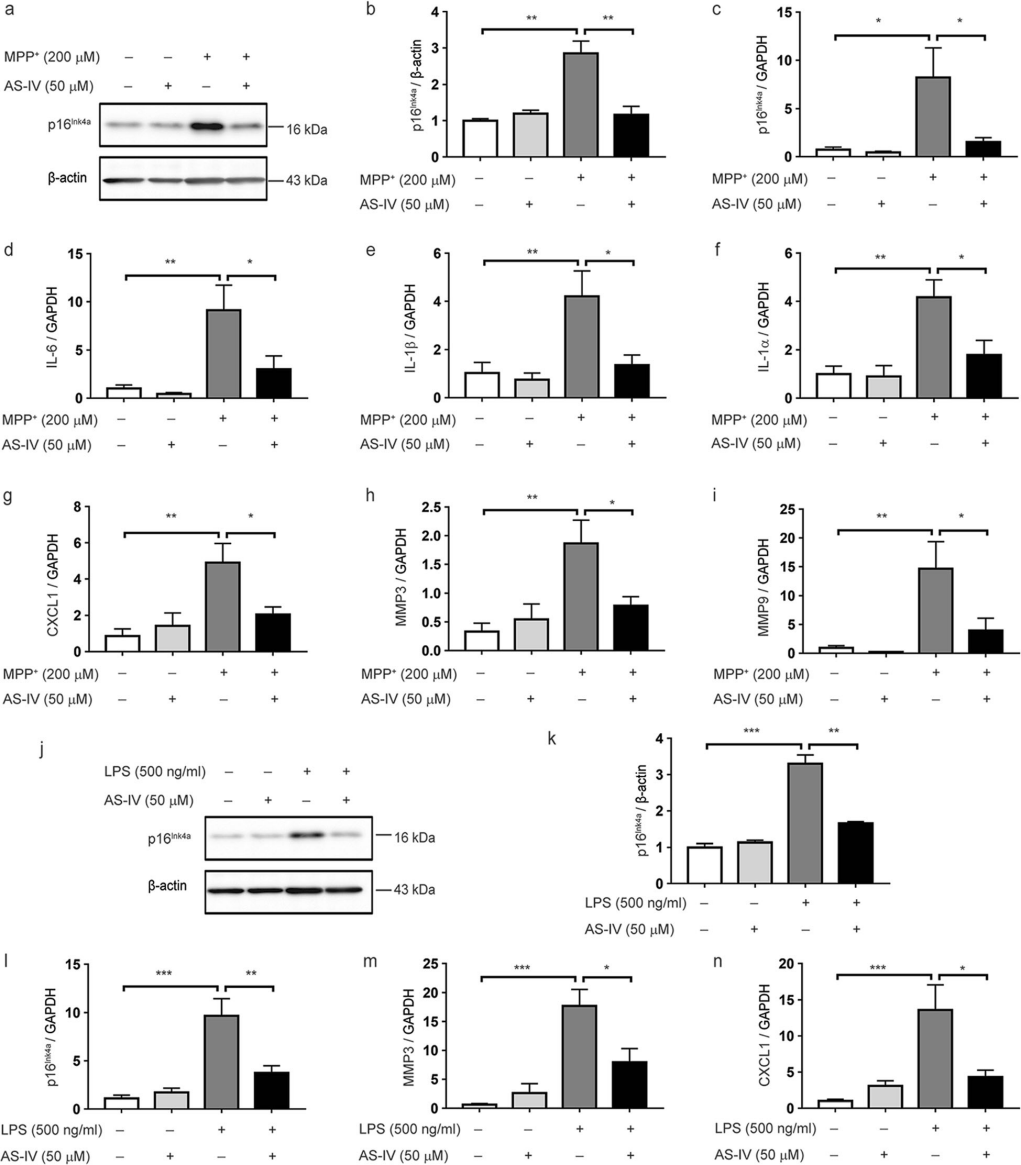
AS-IV suppresses MPP^+^ /LPS-induced senescence of astrocytes. Astrocytes were pretreated with AS-IV (50 μM) for 1 h before MPP^+^ (200 μM) or LPS (500 ng/ml) stimulation for 24 h. **a**, **b** Representative immunoblots (**a**) and quantitative analysis of p16^Ink4a^ (**b**) in astrocytes treated with AS-IV and MPP^+^. Quantified data are normalized to the control group (the control group value is equal to 1). **c**–**i** qPCR of p16^INK4a^ (**c**), IL-6 (**d**), IL-1β (**e**), IL-1α (**f**), CXCL1 (**g**), MMP3 (**h**), and MMP9 (**i**) mRNA levels. **j**, **k** Representative immunoblots (**j**) and quantitative analysis of p16^Ink4a^ (**k**) in astrocytes treated with AS-IV and LPS. Quantified data are normalized to the control group (the control group value is equal to 1). **l**–**n** qPCR of p16^INK4a^ (**l**), MMP3 (**m**), and CXCL1 (**n**) mRNA levels. The data shown are the mean ± SEM from three independent experiments. ^*^p < 0.05, ^**^p < 0.01, ^***^p < 0.001. AS-IV, Astragaloside IV; MPP^+^, 1-methyl-4-phenylpyridinium; LPS, lipopolysaccharide

We also assessed the ability of AS-IV on astrocytes to undergo senescence in response to MPP^+^, the active metabolite of MPTP significantly associated with PD. Cultured astrocytes exposed to MPP^+^ displayed several hallmarks of senescence, including increased levels of p16^Ink4a^ mRNA and protein (Fig. 4a–c), and elevated production of the pro-inflammatory IL-1β, IL-1α, IL-6, Cxcl-1, MMP3, and MMP9, the prominent component of the SASP (Fig. 4d–i), and AS-IV suppressed MPP^+^-induced increase of p16^Ink4a^ and the SASP production. As inflammation plays a critical role in senescence, we used a LPS-induced cell senescence model to verify the effects of AS-IV on astrocyte senescence. Astrocytes cultured with LPS for 24 h also displayed these phenotypes, such as increased p16^INK4a^ mRNA and protein level and production of SASP. Significantly, all LPS-induced senescent phenotypes in astrocytes were abrogated by AS-IV treatment (Fig. 4 h–n). These data demonstrate that AS-IV inhibits MPP^+^/LPS-induced astrocyte senescence.

### AS-IV promotes mitophagy and decreases the mitochondrial ROS production in astrocytes

**Fig. 5:**
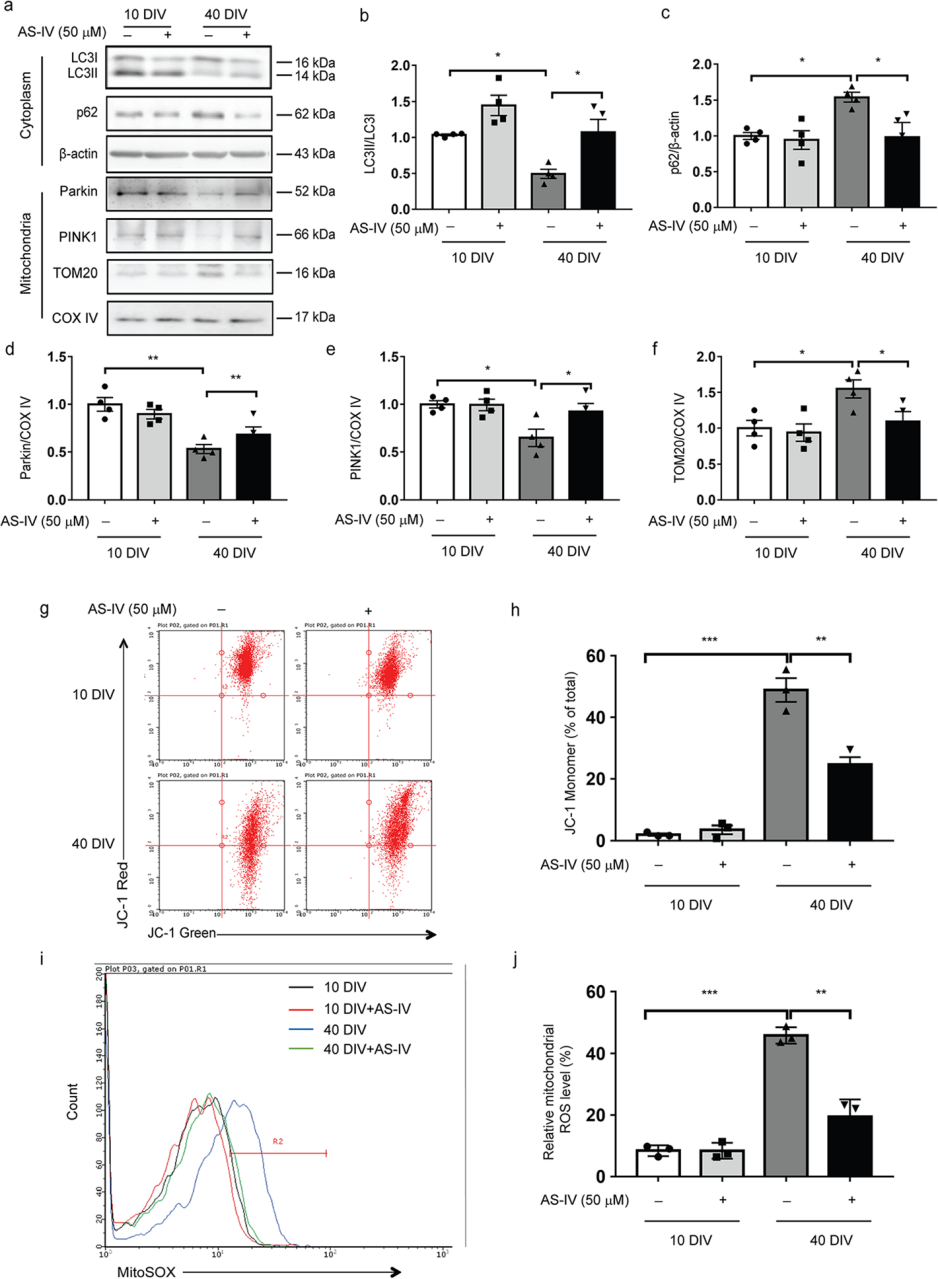
AS-IV promotes mitophagy and decreases the mitochondrial ROS production in astrocytes. Astrocytes were cultured for 10 days or 40 days in vitro (DIV) and then treated with AS-IV (50 μM) for 10 days. **a**–**f** Representative immunoblots (**a**) and quantitative analysis of p62 (**b**) and LC3 (**c**) in the cytoplasm and PINK1 (**d**), Parkin (**e**), and TOM20 (**f**) in the mitochondria of astrocytes. Quantified data are normalized to the control group (the control group value is equal to 1). **g** The scatter plot of the flow cytometry analysis shows the distribution of gating of JC-1 red-positive (aggregates) and JC-1 green-positive (monomers) cell population. **h** The bar graph shows the percentage of JC-1 monomer-positive cells. **i** Astrocytes was stained with MitoSOX and analyzed by flow cytometry. Representative dot plots are shown illustrating the gating of MitoSOX-positive (R2) populations. The percentages of MitoSOX-positive (R2) populations represent relative mitochondrial ROS levels. **j** Quantification of the mitochondrial ROS. The data shown are the mean ± SEM from three independent experiments. ^*^p < 0.05, ^**^p < 0.01, ^***^p < 0.001. AS-IV, Astragaloside IV

Increasing evidence indicates that autophagy has been reported to play crucial roles in cell senescence [19]. To investigate the mechanism by which AS-IV inhibits astrocyte senescence, we examined the levels of autophagy-associated proteins. As shown in Fig. 5a–c, the expression of microtubule-associated protein light chain 3 (LC3)-II was remarkably lower, while the level of p62 was significantly higher in senescent astrocytes than in nonsenescent astrocytes, as measured by immunoblotting, and this was markedly attenuated by AS-IV. Mitophagy is a specialized form of autophagy that plays an essential role in cell senescence. In addition, the expressions of mitophagy-related proteins that included PINK1 and Parkin were notably lower. In contrast, the level of TOM20 was higher in mitochondrial protein extracts from senescent astrocytes than nonsenescent astrocytes, indicating decreased mitophagy flux (Fig. 5d–f). AS-IV treatment could restore the level of mitophagy in senescent astrocytes. More importantly, AS-IV improved the mitochondrial membrane potential, as measured using a fluorescence probe JC-1 assay system (Fig. 5 g, h), and reduced robust mitochondrial ROS production in senescent astrocytes, as measured by mitochondrial superoxide indicator MitoSOX (Fig. 5i, j). These results suggest that AS-IV improves mitochondrial function and decreases mitochondrial ROS generation via promoting mitophagy in senescent astrocytes.

### Inhibition of autophagy reverses the suppressive effect of AS-IV on astrocyte senescence

**Fig. 6:**
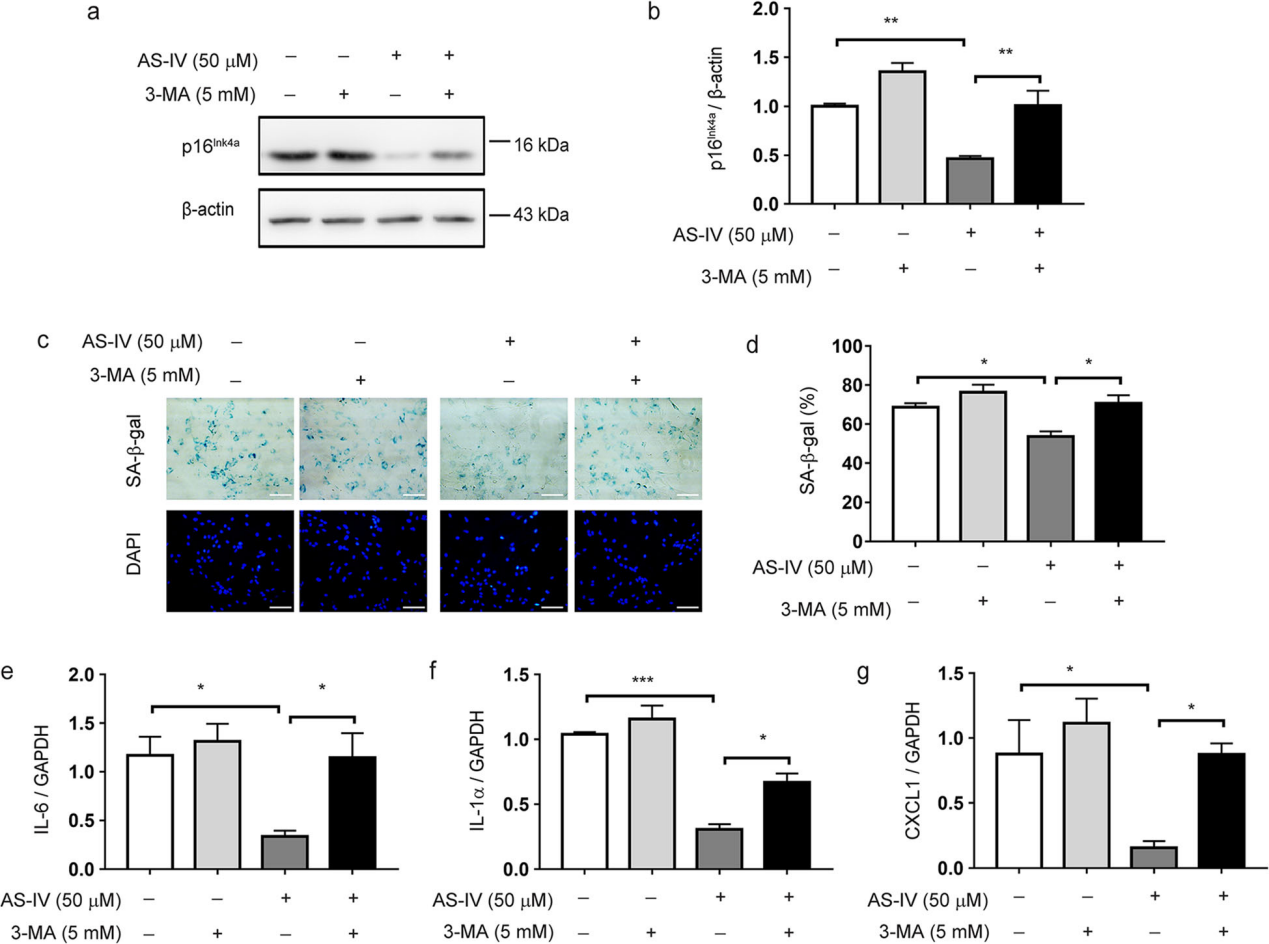
Inhibition of autophagy reverses the suppressive effect of AS-IV on astrocyte senescence. Astrocytes (40 days) were treated with 3-MA (3 mM) for 1 h before AS-IV (50 μM) treatment for 10 days. **a, b** Representative immunoblots (**a**) and quantitative analysis of p16^Ink4a^ (**b**) in astrocytes. Quantified data are normalized to the control group (the control group value is equal to 1). **c, d** Representative images of SA-β-gal activity (**c**) and the percentage of SA-β-gal^+^ cells (**d**). DAPI staining nucleus (blue). Scale bar 100 μm. **e–g** qPCR of IL-6 (**e**), IL-1α (**f**), and CXCL1 (**g**) mRNA levels. The data shown are the mean ± SEM from three to five independent experiments. ^*^p < 0.05, ^**^p < 0.01, ^***^p < 0.001. AS-IV, Astragaloside IV

To test whether autophagy is one of the main mechanisms of AS-IV-mediated astrocyte senescence inhibition, we used the autophagy inhibitor 3-MA to determine if inhibition of autophagy could abolish the suppressive effect of AS-IV on astrocyte senescence. We found that the inhibitory effects of AS-IV on all senescent phenotypes, including downregulated expression of p16^Ink4a^ (Fig. 6a, b), decreased SA-β-gal activity (Fig. 6c, d), and reduced SASP (Fig. 6e–g) in astrocytes, were significantly blocked by 3-MA. These data indicate that AS-IV inhibits astrocyte senescence through promoting autophagy.

## Discussion

The most critical finding presented here is that the small molecule AS-IV inhibits astrocyte senescence. Cellular senescence, an age-associated phenomenon, was first discovered in vitro by extensive culturing of fibroblasts [20]. Emerging evidence displayed that astrocytes from Alzheimer’s disease patients showed increased expression of the senescence markers p21 and p16. Besides, it was demonstrated that astrocytes exhibited eventual growth arrest at late passages of in vitro culturing compared to early passages, indicating replicative senescence from primary astrocytes [21, 22]. Astrocytes have also been documented to undergo stress-induced premature senescence [23–25]. Therefore, astrocytes have been displayed to undergo cellular senescence in vitro due to a variety of factors such as replicative lifespan or external stressors. Resveratrol and a p38MAPK inhibitor could reverse the senescent phenotypes, indicating that senescence has been therapeutically targeted [26, 27]. In the present study, we have found that small molecule AS-IV inhibits senescent astrocyte phenotypes that include increased SA-β-gal activity, elevated expression of the senescence marker p16^Ink4a^, upregulated several SASP factors, and reduced nuclear level of lamin B1, in both extended culture-induced replicative senescence model and MPP^+^/LPS-induced premature senescence model. More importantly, we have also shown that AS-IV treatment reduces the expression of p16^Ink4a^ and increases the nuclear level of lamin B1 in astrocytes in the MPTP-induced PD model. These data indicate that small molecule AS-IV could suppress astrocyte senescence in vitro and PD model mice.

Our study further reveals the molecular mechanism underlying the suppressive effects of AS-IV on astrocyte senescence. Metabolic inflammation, oxidative stress, and mitochondrial dysfunction are believed to be involved in cell senescence [28, 29]. Emerging evidence has revealed a positive correlation between activation of autophagy and longevity [30, 31]. Autophagy is a catabolic degradation system used to degrade and recycle the unnecessary or damaged components of a cell [32]. Increasing evidence shows that autophagy defective is implicated in aging and age-related pathologies. Thus, pharmacologically stimulated autophagy may ameliorate the symptoms of age-associated phenotypes [33, 34]. Mitophagy is a specialized form of autophagy that degraded the damaged and dysfunctional mitochondria [35]. A reduction in mitophagy leads to damaged mitochondria accumulation and oxidative stress, which contribute to cell senescence [36]. In the present study, we have observed the defective mitophagy in senescent astrocytes that included reduced LC3-II expression and elevated p62 expression, decreased PINK1 and Parkin levels, and increased TOM20 expression in mitochondria. The defective mitophagy in senescent astrocytes was reversed by treatment with AS-IV. As a consequence, AS-IV decreases the accumulation of damaged mitochondria and mitochondrial ROS generation in senescent astrocytes. Importantly, inhibition of autophagy abolishes the inhibitory effects of AS-IV on senescent astrocyte phenotypes. These findings indicate that AS-IV inhibits astrocyte senescence by promoting mitophagy. However, it should be further confirmed whether inhibition of autophagy in vivo reverses the suppressive effects of AS-IV on astrocyte senescence and DA neuron degeneration in the MPTP PD mouse model.

**Fig. 7:**
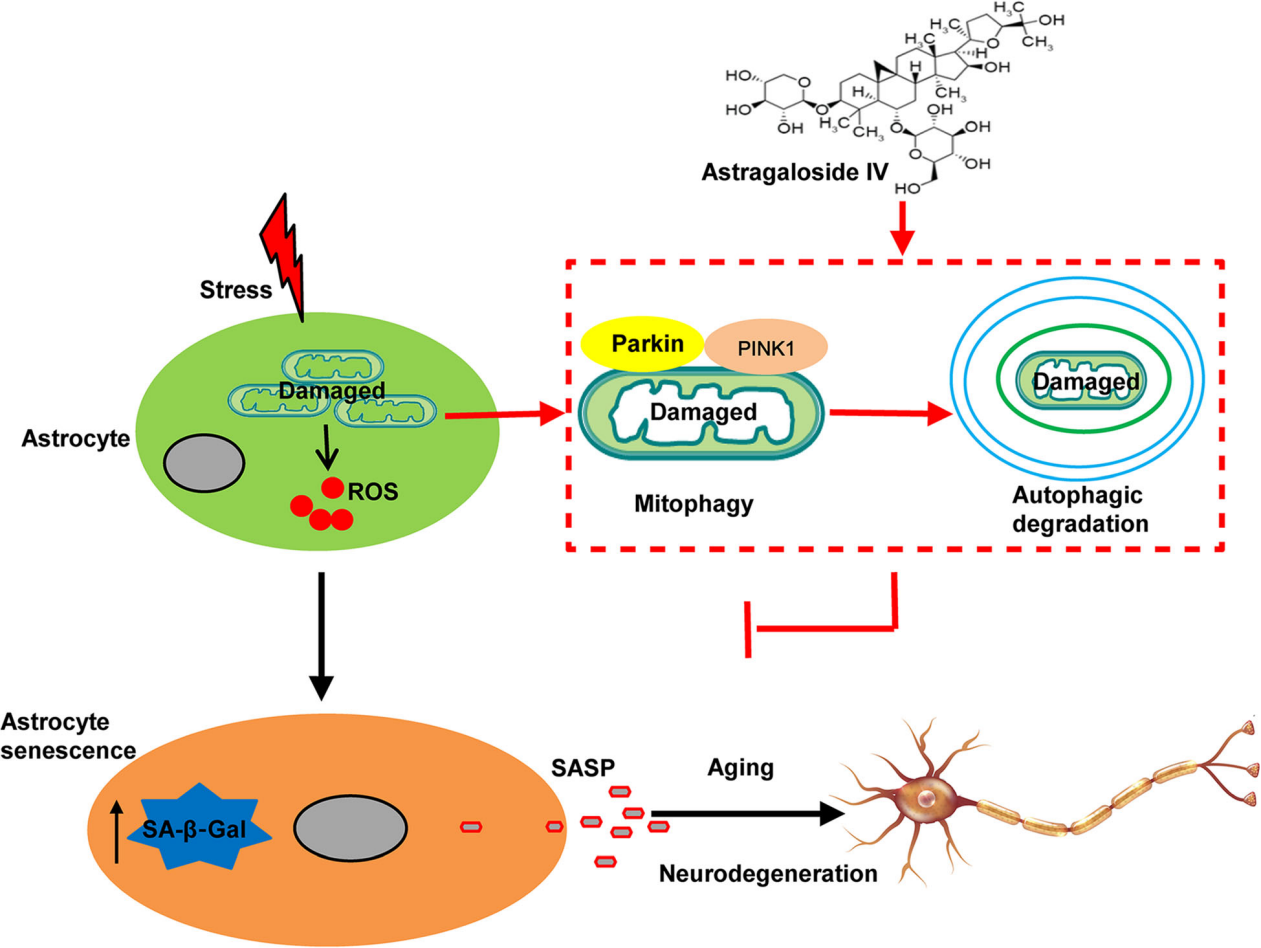
Proposed model depicting AS-IV functions as an autophagy promoter to degrade damaged mitochondria and reduce ROS production, which inhibits astrocyte senescence and, consequently, the neurodegenerative process

Growing evidence indicates that astrocyte senescence plays a vital role in the pathogenesis of PD [37, 38]. Astrocytes provide structural, metabolic, and trophic support to neurons [39, 40]. When astrocytes are dysfunctional, they can impair neuronal function and lead to age-associated neurodegeneration in vivo [2, 41, 42]. Factors secreted by senescent astrocytes had detrimental effects on both cultured human DA neurons and neural progenitor cells [43, 44]. Importantly, it was demonstrated that the levels of senescence markers in astrocytes from PD patients were significantly higher compared to control. Paraquat, an environmental insult significantly associated with PD, caused astrocyte senescence in vitro and in mice. In addition, depletion of senescent astrocytes alleviated PD-like neuropathological phenotypes [11]. In the present study, we have shown that MPTP PD mice had high levels of astrocytes positive for senescence markers, which companied by increased the loss of DA neurons and behavior deficits. AS-IV inhibited astrocyte senescence both in vitro and in mice and rescued DA neuron loss and behavioral deficits in the mouse model of PD. Therefore, it is reasonable to conclude that AS-IV promotes mitophagy, and this enhanced mitophagy decreases the damaged mitochondria accumulation and reduces mitochondrial ROS production in astrocytes, which contributes to the inhibition of astrocyte senescence and SASP factors secretion, thereby attenuating PD pathology (Fig. 7).

## Conclusions

Our findings reveal that AS-IV prevents DA neurodegeneration in PD via inhibition of astrocyte senescence through promoting mitophagy and suggest that AS-IV is a promising therapeutic strategy for the treatment of age-associated neurodegenerative disease such as PD.

## Supplementary information

**Additional file 1:Figure S1** and **S2.**

## Data Availability

All data generated and/or analyzed during this study are included in this article.

## Abbreviations

PD: Parkinson’s disease
AS-IV: Astragaloside IV
LPS: Lipopolysaccharide
MPP^+^: 1-Methyl-4-phenylpyridinium
DA: Dopaminergic
SNc: Substantia nigra compacta
CNS: Central nervous system
MPTP: 1-Methyl-4-phenyl-1,2,3,6-tetrahydropyridine
TH: Tyrosine hydroxylase
GFAP: Glial fibrillary acidic protein
SASP: Senescence-associated secretory phenotype
ROS: Reactive oxygen species
IL-6: Interleukin-6
LC3: Microtubule-associated protein light chain 3
