# Supplementary material for: Astragaloside IV inhibits astrocyte senescence: implication in Parkinson’s disease
Source: J Neuroinflammation. 2020 Apr 6;17:105. doi: 10.1186/s12974-020-01791-8 (PMC7137443; doi:10.1186/s12974-020-01791-8)
Supplement: Supplementary file 1 — Additional file 1:Figure S1 and S2. [file 12974_2020_1791_MOESM1_ESM.docx]

**Flow cytometric analysis of astrocytes at 10 days and 40 days in vitro (DIV) exhibited cell cycles with proportion of cells at G0/G1, S and G2/M phases**


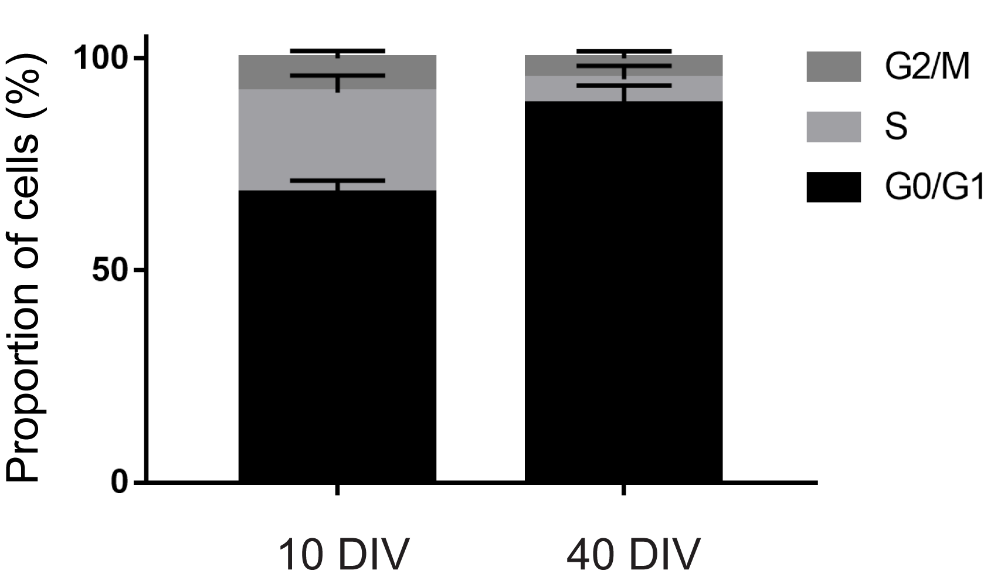


**Fig. s1** **Flow cytometric analysis of astrocytes at 10 days and 40 days in vitro (DIV) exhibited cell cycles with proportion of cells at G0/G1, S and G2/M phases.**

**AS-IV did not affect the activity of MAO-B in the SNc**

**
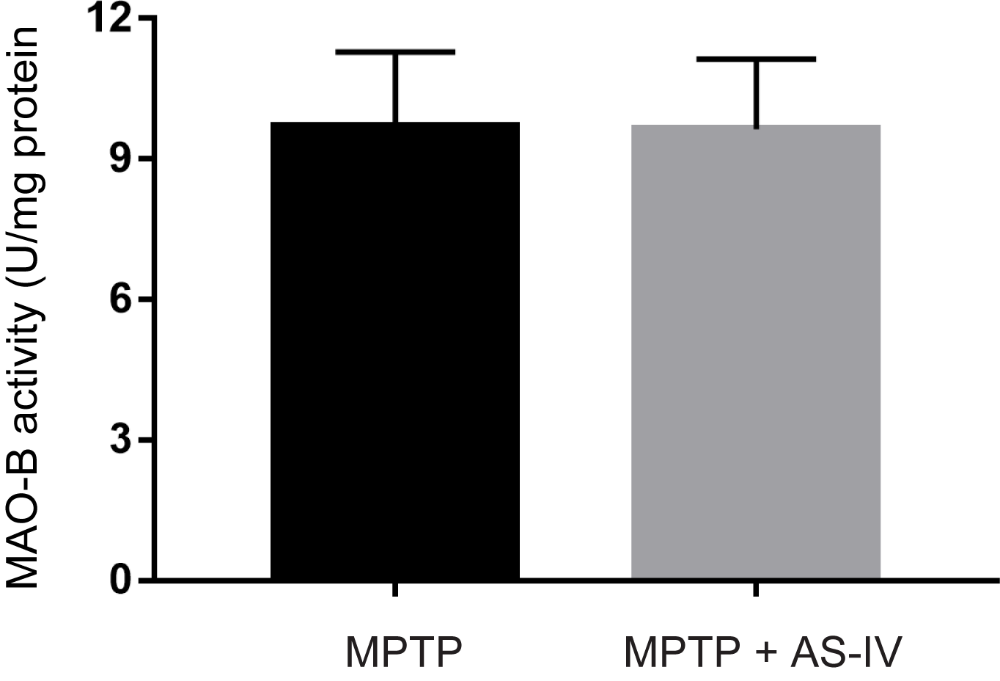
**

**Fig. s2 Elisa analysis of MA0-B activity in the SNc from MPTP-treated mice.**
